# Early and Delayed Impact of Nanosilver on the Glutamatergic NMDA Receptor Complex in Immature Rat Brain

**DOI:** 10.3390/ijms22063067

**Published:** 2021-03-17

**Authors:** Beata Dąbrowska-Bouta, Grzegorz Sulkowski, Mikołaj Sałek, Małgorzata Frontczak-Baniewicz, Lidia Strużyńska

**Affiliations:** 1 Laboratory of Pathoneurochemistry, Department of Neurochemistr, Mossakowski Medical Research Institute, Polish Academy of Sciences, Pawińskiego 5, 02-106 Warsaw, Poland; bbouta@imdik.pan.pl (B.D.-B.); gsulkowski@imdik.pan.pl (G.S.); msalek@imdik.pan.pl (M.S.); 2 Electron Microscopy Platform, Mossakowski Medical Research Institute, Polish Academy of Sciences, Pawińskiego 5, 02-106 Warsaw, Poland; mbaniewicz@imdik.pan.pl

**Keywords:** silver nanoparticles, developmental neurotoxicity, LTP, NMDA receptor, NR2B, cell death pathway

## Abstract

Silver nanoparticles (AgNPs) are the one of the most extensively used nanomaterials. The strong antimicrobial properties of AgNPs have led to their use in a wide range of medical and consumer products. Although the neurotoxicity of AgNPs has been confirmed, the molecular mechanisms have not been extensively studied, particularly in immature organisms. Based on information gained from previous in vitro studies, in the present work, we examine whether ionotropic NMDA glutamate receptors contribute to AgNP-induced neurotoxicity in an animal model of exposure. In brains of immature rats subjected to a low dose of AgNPs, we identified ultrastructural and molecular alterations in the postsynaptic region of synapses where NMDA receptors are localized as a multiprotein complex. We revealed decreased expression of several NMDA receptor complex-related proteins, such as GluN1 and GluN2B subunits, scaffolding proteins PSD95 and SynGAP, as well as neuronal nitric oxide synthase (nNOS). Elucidating the changes in NMDA receptor-mediated molecular mechanisms induced by AgNPs, we also identified downregulation of the GluN2B-PSD95-nNOS-cGMP signaling pathway which maintains LTP/LTD processes underlying learning and memory formation during development. This observation is accompanied by decreased density of NMDA receptors, as assessed by a radioligand binding assay. The observed effects are reversible over the post-exposure time. This investigation reveals that NMDA receptors in immature rats are a target of AgNPs, thereby indicating the potential health hazard for children and infants resulting from the extensive use of products containing AgNPs.

## 1. Introduction

Rapid development of nanotechnology has occurred over the past decade. Silver nanoparticles (AgNPs) are among the nanomaterials which have attracted significant interest, because their unique characteristics make them useful in a wide spectrum of applications. The strong antimicrobial properties of AgNPs are of particular interest resulting in their use in medical and consumer products (for a review see [1]). 

At nano scale, solid materials acquire various altered characteristics, the most important of which is a high surface-to-volume ratio. This feature contributes significantly to the high chemical and biological activities of nanoparticles. Therefore, AgNPs are able to freely cross cellular membranes, accumulate in subcellular compartments and actively interact with biomolecules. 

It has been proven that when AgNPs are present in the circulatory system, they enter the brain of rodents via the blood–brain barrier [2] and accumulate to a greater extent in the nervous tissue of immature animals relative to adult animals if subjected to a low-dose exposure [3]. There is also evidence that neurons are more vulnerable than glial cells to the presence of AgNPs and their toxic effects [3].

It has been reported that about 80–90% of all neurons in the brain are glutamatergic neurons that use glutamate as a neurotransmitter [4]. Excitatory neurotransmission underlies crucial aspects of our behavior, including learning and memory formation. Glutamatergic synapses are asymmetric, being composed of a thin presynaptic membrane and a thick postsynaptic part that is referred to as postsynaptic density (PSD) [5]. PSD is a protein complex consisting of hundreds of proteins, including scaffolding proteins, signaling enzymes, cytoskeletal proteins and receptors among which ionotropic glutamatergic N-methyl-D-aspartate (NMDA) and α-amino-3-hydroxy-5-methyl-4-isoxazole propionic acid (AMPA) receptors are major components [6]. The NMDA receptor functions as a ligand-gated ion channel that is widely expressed in all brain areas that mediate inward currents of Ca^2+^ and Na^+^ ions and outward currents of K^+^ ions. This heterotetrameric receptor consists of two obligatory GluN1 (NR1) subunits and two GluN2 (NR2) subunits of different subtypes (A–D) in various combinations, which determine the functional diversity of the receptor [7]. Anchored at the postsynaptic plasma membrane, the NMDA receptor binds to a variety of proteins, forming a multiprotein complex. The NMDA receptor is associated with scaffolding proteins that help to stabilize the receptor, such as PSD-95, GKAP, SHANK or synaptic GTPase activating protein (SynGAP), a negative regulatory factor of Ras small GTPase that is also present in a large quantity in PSDs [6]. SynGAP has been shown to interact preferentially with GluN2B containing the NMDA receptor [8]. In turn, PSD-95 is a pivotal protein in the complex that interacts with many different effector enzymes, such as SynGAP, kinases, phosphatases and signaling molecules including Ca^2+^/calmodulin-dependent kinase (CaMK II), neuronal nitric oxide synthase (nNOS), adenylate cyclase (AdCyc) and others [5]. 

In addition to providing vital roles in brain function, the NMDA receptor may also substantially contribute to the brain pathologies underlying a variety of major nervous system disorders, including neurodegenerative diseases, ischemia, schizophrenia and depression [9]. In pathological states, altered expression/function of the NMDA receptor may either result in inhibition in neuronal circuitry or in overactivation leading to neuronal death in the mechanism of excitotoxicity [9,10]. 

Our previous in vitro study confirmed the involvement of the glutamatergic NMDA receptor type in neurotoxic effects evoked by AgNPs in cerebellar granule cells (CGC) [11]. Our results revealed that AgNPs intensify the entry of extracellular calcium into cultured neurons. This phenomenon is completely abolished by addition of 0.5 µM MK-801, a noncompetitive inhibitor of the NMDA receptor. Increased calcium uptake further induces an intracellular calcium imbalance followed by a decrease of mitochondrial membrane potential and parallel production of reactive oxygen species (ROS). All of these effects are also fully or partially reversible by administration of MK-801. All of these abnormalities lead to death of granule cells, indicating that in vitro AgNP-induced neurotoxic effects are almost partially mediated by the glutamatergic NMDA receptor. However, a role of the NMDA receptor in the mechanisms of AgNP-induced neurotoxicity has been not yet confirmed in animal models of exposure. 

Therefore, in the present work, we test a hypothesis concerning the involvement of the glutamatergic NMDA receptor in neurotoxicity of AgNPs in immature rats. This hypothesis is important because immature organisms are more vulnerable to various toxins than adults due to immaturity of metabolic and detoxifying functions of vital organs [12]. Furthermore, the NMDA receptor is involved in cognitive functions and the mechanisms of neurotoxicity of AgNPs in the developing brains of immature organisms remains largely unknown.

In the present study, we examine whether prolonged exposure to a low dose of AgNPs (0.2 mg/kg b.w.) influences the expression of the NMDA receptor multiprotein complex with particular emphasis on NMDA receptor subunits (NR1 and NR2B), PSD-95, SynGAP and nNOS. Analyses of mRNA and protein expression are combined with ultrastructural analysis of synapses in brains of exposed animals and functional characteristics of the NMDA receptor. Moreover, the observed effects are compared to the effects of ionic silver (administered as Ag citrate) over time, i.e., early (postnatal day 35; PND 35) and late (PND 90) after exposure.

## 2. Results

### 2.1. Silver Accumulates in the Brain of Exposed Rats and Induces Ultrastructural Alterations in Synapses Early after Exposure

**Figure 1:**
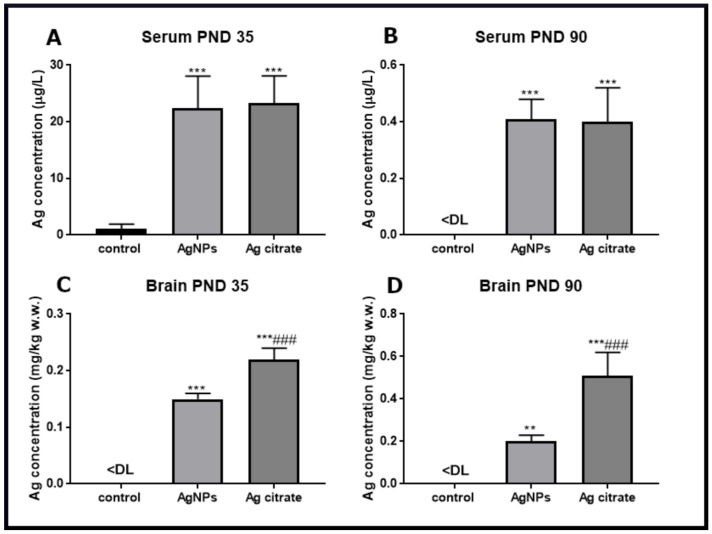
Silver concentrations in selected tissue compartments of AgNP- and Ag citrate-exposed immature rats measured by ICP-MS method shortly (PND 35) and late (PND 90) after exposure. Silver concentrations in the serum in PND 35 (**A**) and PND 90 (**B**); silver concentrations in the brain in PND 35 (**C**) and PND 90 (**D**). Values are means ± SD from three independent samples; *** *p* < 0.001 vs. control, ### *p* < 0.001 vs. AgNPs (one-way ANOVA with post-hoc Tukey’s test); DL—detection limits: for serum = 0.190 µg/L, for solid tissue = 0.011 mg/kg w.w.

Silver, administered orally as AgNPs or in the ionic form, is adsorbed equally from the gastrointestinal tract into the blood compartment where its concentration in the serum reaches about 23 µg/L just after the three-week exposure (PND 35) (Figure 1A). Following the post-exposure time, the concentrations of silver decline significantly in the serum of both of the silver-treated groups and almost approach the control level at PND 90 (Figure 1B). 

Silver accumulates in brains of animals at concentrations of about 0.15 mg/kg and 0.23 mg/kg for AgNP-treated and Ag citrate-treated rats, respectively (Figure 1C), whereas in control brain tissue, silver is below the detection limit. With post-exposure time, the concentration of silver persists in brain tissue indicating the tendency to accumulate in this organ (Figure 1D). Time-dependent deposition of silver in brain is significantly higher for ionic silver than for its particulate form (*p* < 0.001 vs. AgNPs). 

**Figure 2:**
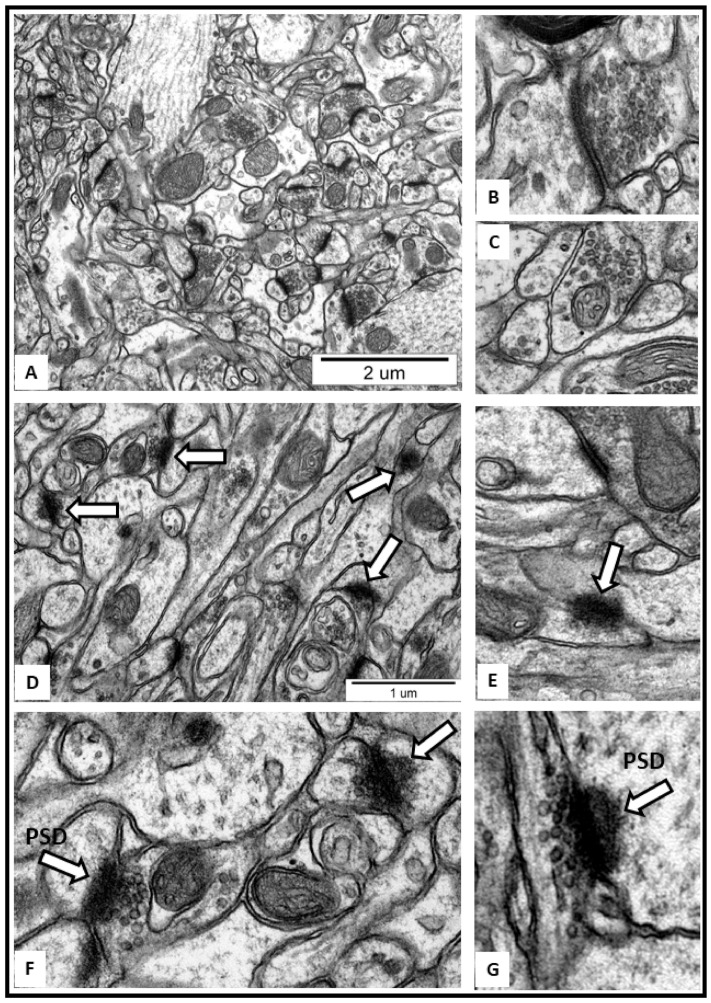
TEM images from brain tissue of control (**A**–**C**) and AgNP/Ag citrate-exposed (**D**–**G**) rats. (**A**) Proper ultrastructure of neuropil (**A**) with normal appearance of synapses; (**B**,**C**) well-defined pre- and post-synaptic membranes with thin postsynaptic densities (PSDs) at higher magnification. (**D**) ultrastructural alterations in synapses in a form of blurred synaptic cleft. Synaptic membranes are not clearly visible, and PSDs are abnormally thickened (arrows); (**E**–**G**) higher magnifications of distinct synapses showing ultrastructural details, including changed PSDs. Images are representative for three animals in each group.

AgNPs and Ag^+^ present in brains of immature rats influence the ultrastructure of synapses causing them to have an altered appearance relative to synapses of control rat brain tissue. Electron microscopy analysis of brain samples obtained from control rats reveals the proper structure of neuropil wherein well-defined synapses exhibit a proper distribution of synaptic vesicles and a narrow synaptic cleft. All membranes contributing to the structure of the synapse are readily distinguishable and the postsynaptic densities are prominent and clearly stained (Figure 2A–C). In electronograms obtained from AgNP-treated rat brain tissue, a large proportion of the synapses exhibit blurred ultrastructure. The synaptic membranes are difficult to identify therefore the synaptic cleft is not clearly visible. The postsynaptic densities (PSDs) are irregular and excessively thickened (Figure 2D–G). 

Abnormal synapses are observed in similar numbers in different brain regions, i.e., cerebral cortex and hippocampus and are present in similar amounts in both of the silver-treated groups. Late after exposure, the changes described in synapses are much less visible in both silver-treated groups as compared to respective 35 PND groups.

### 2.2. Silver Influences the Expression of Selected Proteins Related to the NMDA Receptor Complex

Time-dependent profiles of changes in the expression of several proteins of the NMDA receptor complex such as receptor subunits GluN1 GluN2B, as well as PSD-95, SynGAP and nNOS were determined at the level of mRNA and protein.

**Figure 3:**
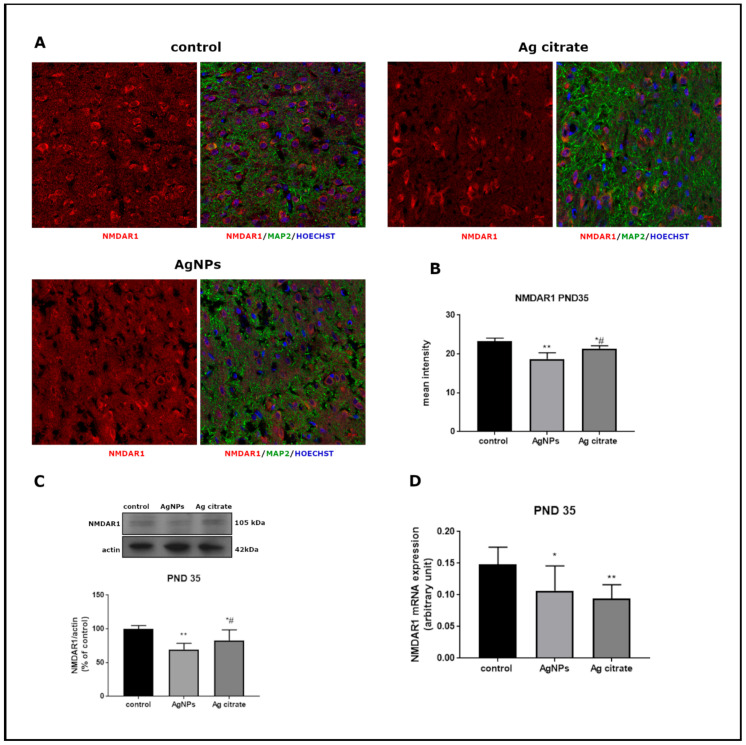
The expression of GluN1 (NR1) subunit of NMDA receptor in the brain of control and AgNPs/Ag citrate-exposed immature rats in PND 35. (**A**) Representative confocal images of the cerebral cortex; triple immunostaining of GluN1 (red) with anti-MAP antibody (green) and Hoechst to visualize cell nuclei (blue); scale bars indicate 20 µm. (**B**) The mean intensity of the fluorescence; data are means ± SD from 9–10 sections from three distinct brains in each group, * *p* < 0.05 and ** *p* < 0.01 vs. control, # *p* < 0.05 vs. AgNPs. (**C**) Relative expression of GluN1 protein measured against β-actin as an internal standard. Representative immunoblot and graph illustrating the mean results obtained using four distinct animals. (**D**) Expression of GluN1 mRNA determined by qPCR and normalized against actin. Data in (**C**,**D**) are means ± SD from four experiments, * *p* < 0.05, ** *p* < 0.01 vs. control, # *p* < 0.05 vs. AgNPs (one-way ANOVA followed by Tukey’s multiple comparison test).

**Figure 4:**
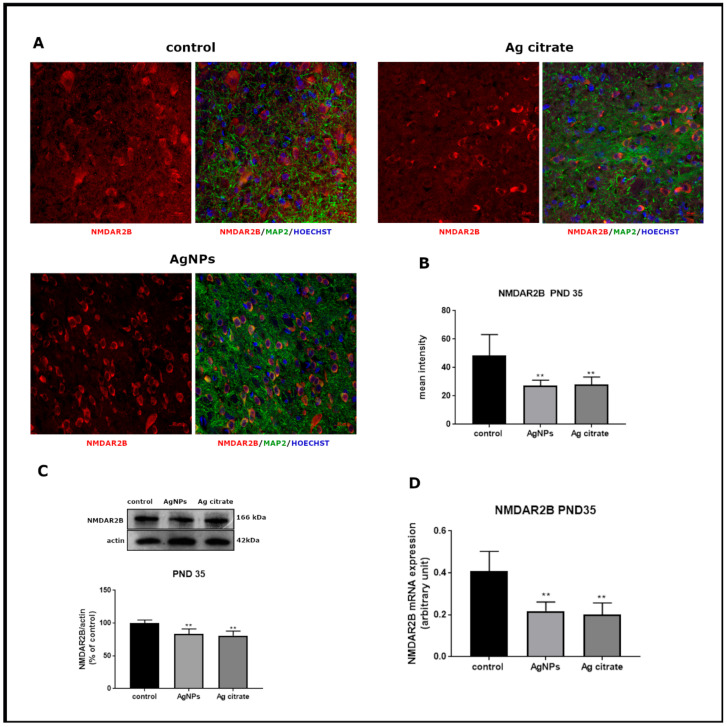
The expression of GluN2B (NR2) subunit of NMDA receptor in the brain of AgNPs/Ag citrate-exposed immature rats in PND 35. (**A**) Representative confocal images of cerebral cortex; triple immunostaining of GluN2B (red) with anti-MAP antibody (green) and Hoechst to visualize cell nuclei (blue); scale bars indicate 20 µm. (**B**) The mean intensity of the fluorescence; data are means ± SD from *n* = 9–10 sections from three distinct brains in each group, ** *p* < 0.01 vs. control. (**C**) Relative expression of GluN2B protein measured against β-actin as an internal standard. Representative immunoblot and graph illustrating the mean results obtained using four distinct animals; ** *p* < 0.01 vs. control (**D**) Expression of GluN2B mRNA determined by qPCR and normalized against actin. Data are represented as means ± SD from four experiments, ** *p* < 0.01 significantly different vs. control (one-way ANOVA followed by Tukey’s multiple comparison test).

Significant alterations were revealed in the relative expression of both mRNA and protein of GluN1 subunit in forebrains of AgNP-treated rats just after exposure (PND 35) (*p* < 0.05 and *p* < 0.01 vs. control, respectively) (Figure 3). Substantial changes were also noted in the mRNA (*p* < 0.01) and protein (*p* < 0.05) expression of GluN2B relative to controls (Figure 4). A similar trend of changes was observed regardless the form of administered silver. 

Immunohistochemical analysis confirmed lower expression of both subunits. A significant decrease in GluN1 (Figure 3A,B) and GluN2B (Figure 4A,B) immunoreactivity was found to be present in both silver-treated groups relative to control. Similar differences in the intensity of immunofluorescence were observed in both examined brain region, i.e., the cerebral cortex and hippocampus.

**Figure 5:**
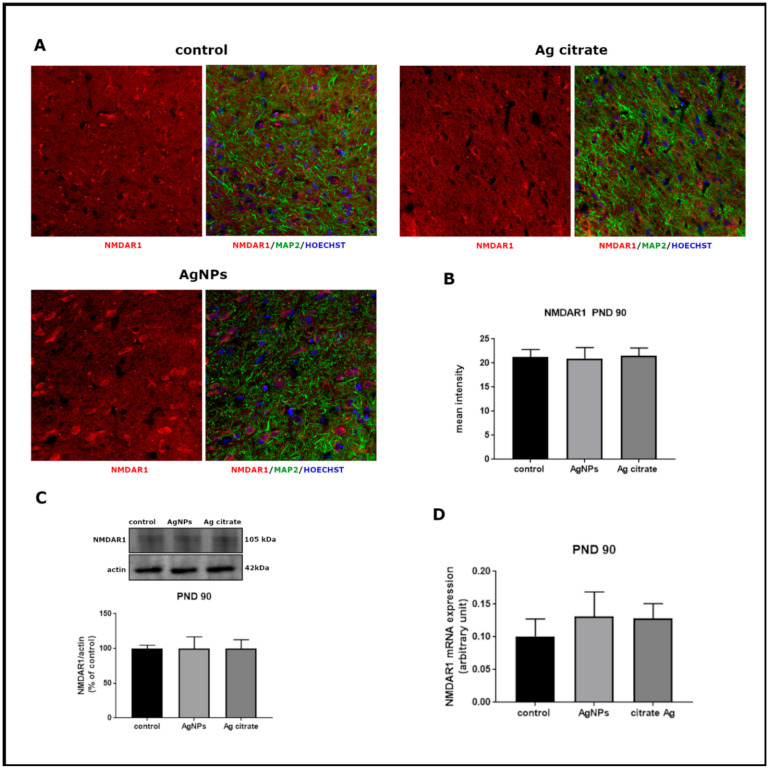
The expression of GluN1 (NR1) subunit of NMDA receptor in the brain of AgNPs/Ag citrate-exposed immature rats in PND 90. (**A**) Representative confocal images of cerebral cortex; triple immunostaining of GluN1 (red) with anti-MAP antibody (green) and Hoechst to visualize cell nuclei (blue); scale bars indicate 20 µm. (**B**) The mean intensity of the fluorescence; data are means ± SD from *n* = 9–10 sections from three distinct brains in each group; there are no significant differences between groups. (**C**) Relative expression of GluN1 protein measured against β-actin as an internal standard. Representative immunoblot and graph illustrating the mean results obtained using four distinct animals. (**D**) Expression of GluN1 mRNA determined by qPCR and normalized against actin. Data are represented as means ± SD from four experiments, there are no statistical differences between groups (one-way ANOVA followed by Tukey’s multiple comparison test).

**Figure 6:**
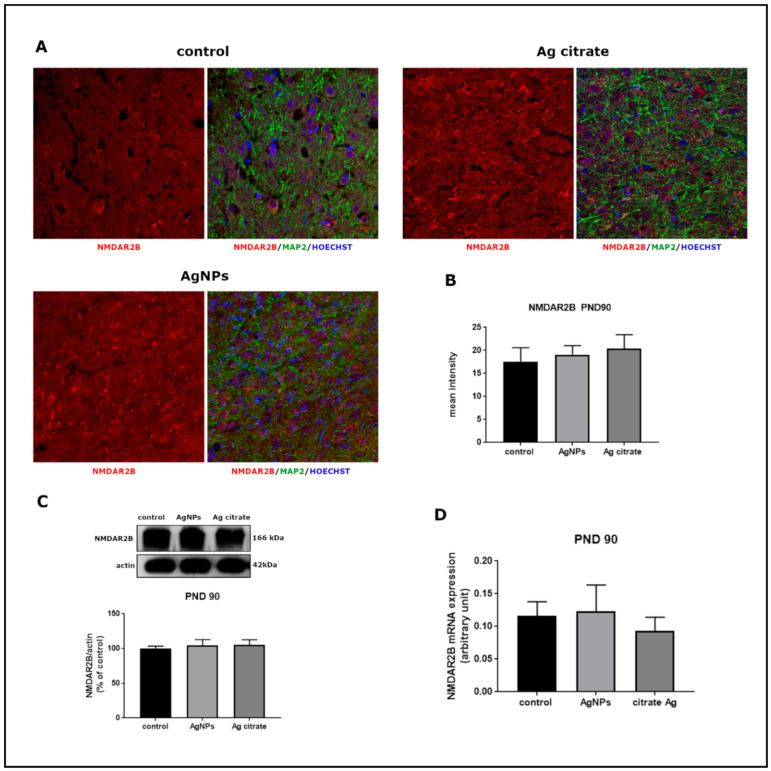
The expression of GluN2B (NR2) subunit of NMDA receptor in the brain of AgNPs/Ag citrate-exposed immature rats in PND 90. (**A**) Representative confocal images of cerebral cortex; triple immunostaining of GluN1 (red) with anti-MAP antibody (green) and Hoechst to visualize cell nuclei (blue); scale bars indicate 20 µm. (**B**) The mean intensity of the fluorescence; data are means ± SD from *n* = 9–10 sections taken from three distinct brains in each group; there are no significant differences between groups. (**C**) Relative expression of GluN2B protein measured against β-actin as an internal standard. Representative immunoblot and graph illustrating the mean results obtained using four distinct animals. (**D**) Expression of GluN2B mRNA determined by qPCR and normalized against actin. Data are represented as means ± SD from four experiments, there are no significant differences between groups (one-way ANOVA followed by Tukey’s multiple comparison test).

Changes in the expression of both receptor subunits were not visible late after exposure (PND 90). The levels of mRNA, proteins, and the intensity of immunoreactivity returned to the levels observed in the control group (Figure 5 and Figure 6). 

**Figure 7:**
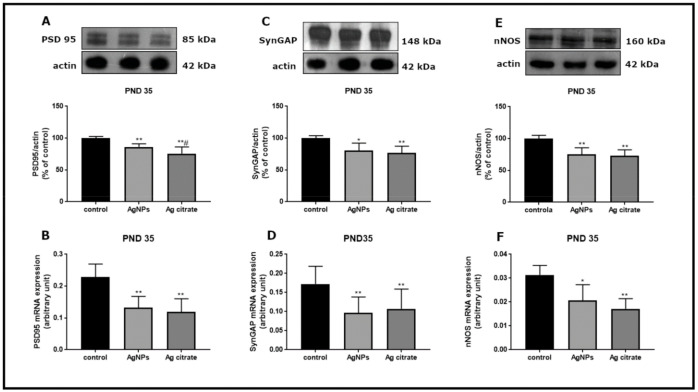
The expression of selected PSD-related proteins in forebrains of AgNPs/Ag citrate-exposed immature rats early (PND 35) after exposure. Representative immunoblots and graphs indicating relative expression of respective proteins and their mRNAs: PSD95 (**A**,**B**), SynGAP (**C**,**D**) and nNOS (**E**,**F**). Data are means ± SD from four independent experiments; * *p* < 0.05, ** *p* < 0.01 vs. control, # *p* < 0.05 vs. AgNPs (one-way ANOVA followed by Tukey’s multiple comparison test).

**Figure 8:**
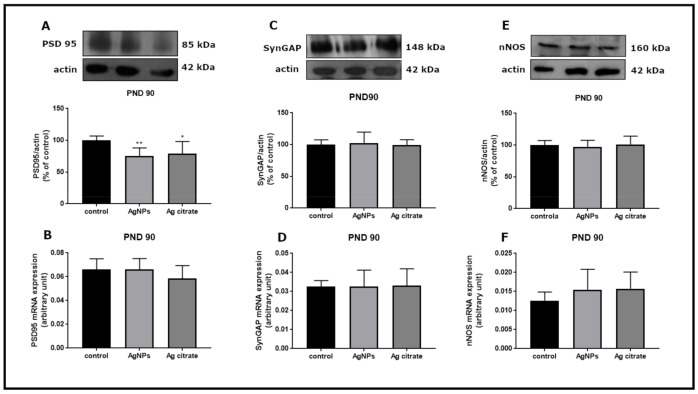
The expression of selected PSD-related proteins in forebrains of AgNPs/Ag citrate-exposed immature rats late (PND 90) after exposure. Representative immunoblots and graphs indicating relative expression of respective proteins and their mRNAs: PSD95 (**A**,**B**), SynGAP (**C**,**D**) and nNOS (**E**,**F**). Data are means ± SD from four independent experiments; * *p* < 0.05, ** *p* < 0.01 vs. control (one-way ANOVA followed by Tukey’s multiple comparison test).

Apart from alterations in the NMDA receptor subunits, down-regulation of genes of selected proteins which are crucial components of the NMDA receptor complex was also noted in both of the silver-exposed groups. PSD-95 mRNA and protein levels are significantly lower relative to respective controls (*p* < 0.01) (Figure 7A,B). Levels of SynGAP (Figure 7C,D) and nNOS (Figure 7E,F) mRNA and proteins are also significantly lower. This trend of changes was maintained late after exposure (PND 90) for PSD-95 exclusively (Figure 8). 

### 2.3. Silver Down-Regulates the nNOS-NO-cGMP Pathway

**Figure 9:**
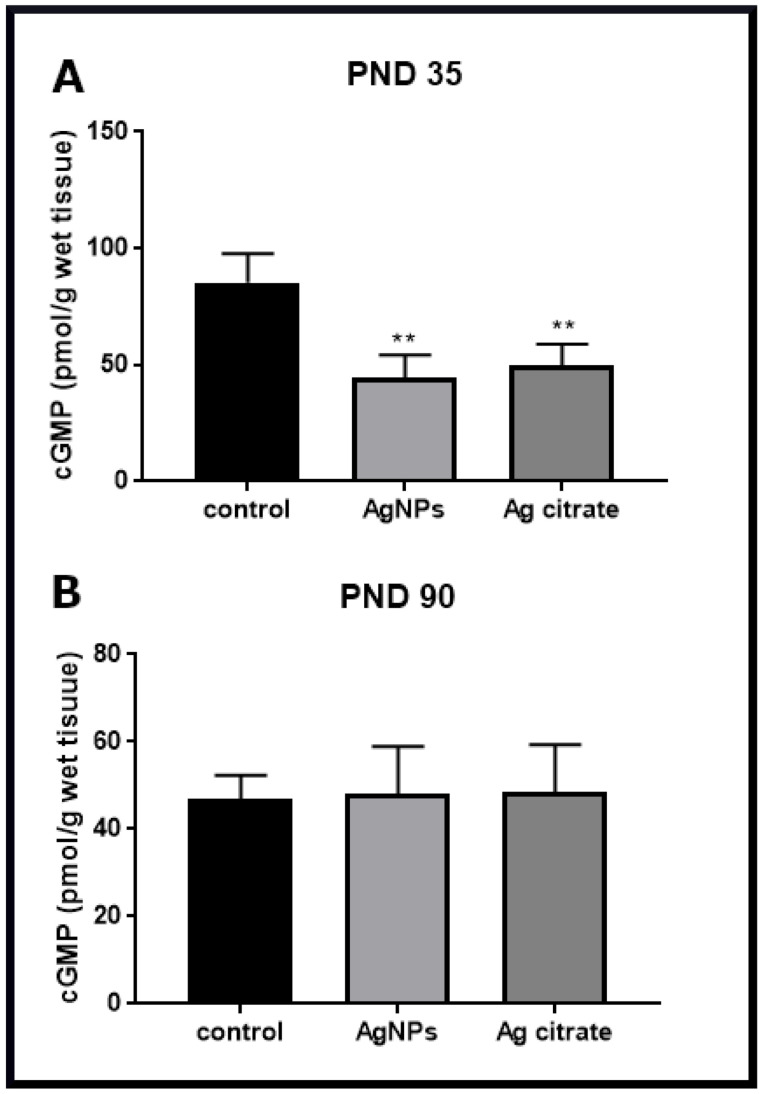
The concentration of cGMP in forebrain samples taken from control, AgNP- and Ag citrate-exposed rats at PND 35 (**A**) and PND 90 (**B**). Data are means ± SD from three independent experiments, ** *p* < 0.01 vs. control (one-way ANOVA followed by Tukey’s multiple comparison test).

In parallel with the analysis of the expression of GluN1 and GluN2B subunits of the NMDA receptor, we examined the expression of nNOS which is a key enzyme producing nitric oxide (NO), a diffusible signaling molecule whose downstream action should increase production of cGMP. As mentioned above, the level of nNOS mRNA expression and the relative concentration of nNOs protein are markedly reduced after exposure to AgNPs or Ag^+^ (Figure 7E,F). Furthermore, a remarkable decrease in the level of cGMP by about 50% relative to control was observed, indicating down-regulation of this signaling pathway (Figure 9). This silver-evoked effect was not present at PND 90. 

### 2.4. Silver Influences the Binding of Ligands to the NMDA Receptor

**Figure 10:**
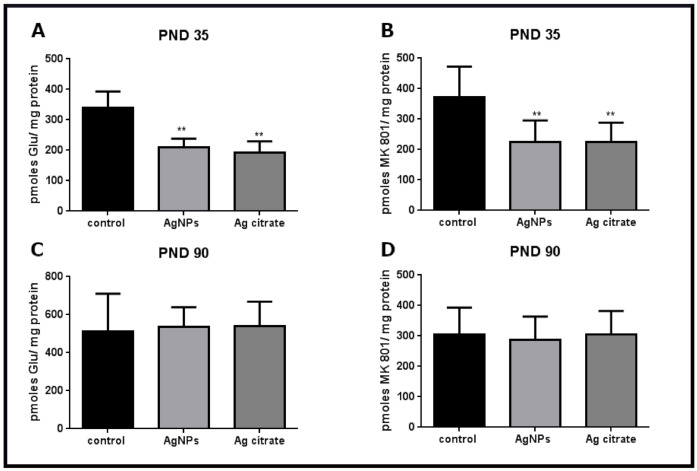
The specific binding of radioligands to the membranes isolated from the forebrains of control, AgNP- and Ag citrate-exposed rats. Binding of the agonist [^3^H]glutamate early after exposure at PND 35 (**A**) and late at PND 90 (**C**). Binding of the selective blocker of NMDA receptors [^3^H]MK-801 early at PND 35 (**B**) and late at PND 90 after exposure (**D**). Data are means ± SD from three independent experiments, ** *p* < 0.01 vs. control (one-way ANOVA followed by Tukey’s multiple comparison test).

Radioligand binding assay can quantify and qualitatively characterize receptors. Saturation binding studies were performed using several concentrations of unlabeled ligands to determine the number of binding sites (Bmax), i.e., the density of receptors. In the presence of AgNPs/Ag^+^ in rat brain tissue, binding of [^3^H]glutamate to the isolated brain membranes was reduced by about 40% relative to control (*p* <0.01) early after exposure (Figure 10A). Since glutamate does not specifically bind to NMDA-type glutamate receptors and, as an agonist, may label different populations of glutamatergic receptor, we additionally measure NMDA receptor density in the presence of MK-801, a selective and irreversible blocker of the NMDA receptor. The binding of [^3^H]MK-801 was found to be diminished by about 46% (*p* < 0.01) (Figure 10B), indicating depletion of binding sites to a similar extent. These results suggest that the NMDA receptor is a specific target of AgNPs/Ag^+^. The effect of the reduction of receptor’s density was found to be reversible with time. No changes relative to control were observed late after exposure at PND 90 in the radioligand binding assays (Figure 10C,D). 

As described in Materials and Methods, we used animals of both sexes in the experiment. Therefore, much attention has been paid to potential gender-related differences while analyzing the results. However, we did not observe statistically significant differences between males and females in any of the analyses. 

## 3. Discussion

Previous studies on the mechanisms of AgNP neurotoxicity in animal models revealed deleterious effects in neurons such as necrotic cell death [13], degeneration of synapses [2] and ultrastructural and biochemical markers of endoplasmic reticulum (ER) stress [3,14]. Likewise, down-regulation of EAAC1 glutamate transporter, a neuronal excitatory amino acid carrier, has been reported in immature rats exposed to AgNPs [3] whose role is connected, among other things, with protection against oxidative stress [15] and the processes of learning and memory formation [16]. 

Since a vast majority of neurons are of glutamatergic activity and because glutamate receptors play pivotal roles in the function of the central nervous system (CNS), it would be helpful to determine whether AgNPs influence the expression and function of the NMDA receptor, which is the main ionotropic type of glutamate receptor known to be involved in physiological and pathological processes in the CNS. Alterations in expression/activity of the NMDA receptor may result in detrimental effects. Hyper-functional receptors cause cell death whereas hypo-functional receptors change the excitation/inhibition balance in the neuronal network [9]. 

To address the potential involvement of the NMDA receptor in AgNP-evoked neurotoxicity, we designed an animal model of early postnatal exposure to a low dose of AgNPs/Ag^+^ (0.2 mg/kg b.w.). This experimental model was chosen for two reasons: (i) research on nanoparticulate silver in immature organisms is rare, and (ii) the NMDA receptor significantly contributes to the proper formation, maturation and functioning of developing synapses [17].

### 3.1. AgNPs/Ag^+^ Alters the Ultrastructure of the Synapses in Developing Brain Tissue of Exposed Rats

While examining the glutamatergic synapses using TEM, a thick electron-dense structure at the postsynaptic membranes, which is known as postsynaptic density (PSD), is typically visible. This structure consists of a core layer and a pallium that becomes more prominent during synapse stimulation, reflecting enhanced levels of several proteins accumulating reversibly therein [18]. Such PSD thickening has previously been described under ischemic conditions [19], as well as following LTP induction [20]. 

Our TEM results revealed abnormally increased electron density at the postsynaptic membrane. This could be regarded as a result of strongly enhanced synaptic activity due to over-activation of the NMDA receptors responsible for morphological and biochemical modifications at the PSDs. However, it was observed that synapses simultaneously acquire the specific blurred structure with indistinct synaptic membranes, particularly the postsynaptic membrane. Hence, ultrastructural characteristics suggest disturbances either at the levels of PSD-related proteins or in their redistribution, which accompanies activation of the synapse. Interestingly, measurements of selected key proteins within the PSD, such as GluN1, GluN2B, PSD-95 and SynGAP, revealed their diminished expression. This does not explain the thickening of the postsynaptic part of the synapse. A possible explanation is that exposure to AgNPs/Ag^+^ influences the protein–protein interactions within the PSD structure altering the pattern of protein composition upon activity-mediated redistribution. This likely occurs as a result of silver ions reacting with protein SH groups and generating significant conformational and functional alterations. In the case of particulate silver, direct dynamic interactions with different proteins are possible which tend to create reversible or irreversible protein coronas [21], thereby temporarily modulating the composition of protein-enriched PSDs. Such compositional changes may result in subsequent alterations in the binding affinity for the compounds used for tissue processing in TEM analysis, thereby explaining the observed blurred pattern of the synapse. However, regardless the mechanisms underlying the alterations of the ultrastructural characteristics of the synapse, silver unequivocally causes a decrease in expression of postsynaptic proteins. 

### 3.2. NMDA Receptor-Dependent Cell-Death Pathway in Silver-Exposed Rat Brains 

Excessive overactivation of the NMDA receptor triggers molecular events leading finally to excitotoxic neuronal cell damage, which is a common molecular component of many neurological disorders. One of the intracellular signaling cascades activated by the influx of Ca^2+^ by the opened NMDA receptor channel is a cell-death pathway mediated by protein complex GluN2B-PSD-95-nNOS [22]. Scaffolding protein PSD-95, which is present at postsynaptic densities, binds the GluN2B subunit and neuronal nitric oxide synthase (nNOS) thereby facilitating interactions between these two proteins and final generation of nitric oxide (NO) by nNOS [23]. NO released from nerve endings stimulates the activity of soluble guanylate cyclase (GC) resulting in an increase in production of cGMP. As a diffusible signaling molecule, NO is physiologically involved in the synaptic plasticity but under pathological conditions of excitotoxicity gains cytotoxic properties [24]. Both nNOS and NO have long been recognized as having major roles in CNS dysfunction and diseases such as stroke, neurodegenerative disorders, psychiatric disorders and chronic pain [25,26].

All elements of the cell-death protein complex which were investigated by us are down-regulated under conditions of a low-dose AgNPs/Ag^+^ exposure. PSD-95 appears to be particularly important in this context, as it is required to efficiently couple the NMDA receptor-gated calcium influx which is necessary for nNOS activation [27,28] by binding to the N-terminal region of nNOS [29].

Diminished mRNA and protein expression of GluN2B, PSD-95 and nNOS indicates targeting of this signaling pathway by silver leading to diminished production of toxic NO, which we identified by measuring cGMP levels. NO generated by nNOS acts as a signaling molecule leading to the formation of cGMP in the nNOS-NO-cGMP molecular pathway. The decreased level of cGMP indicates that this chain of reactions is inhibited in brain tissue in the presence of AgNPs/Ag^+^. It is likely that AgNPs inhibit protein interactions within the NMDA receptor complex, thereby inhibiting downstream events. 

Recent evidence suggests that disrupting or inhibiting this cell-death complex may be an effective neuroprotective strategy [23] which, if applied in animal models of ischemia or stroke, would significantly reduce excitotoxic brain damage [28,30]. Moreover, according to the “subunit/localization” hypothesis [31], signaling via GluN2B-containing NMDA receptors, located mainly extrasynaptically, largely contributes to NMDA receptor-induced cell death [10].

Considering our results against the background of the existing data, we can state that AgNPs/Ag^+^ present in brains of developmentally exposed rats do not evoke the excitotoxicity-like phenomenon mediated by overactivation of NMDA receptors. Our observations made by using an animal model of exposure are counter to the results previously reported in vitro obtained by using AgNP-treated CGC cultures. As mentioned in the Introduction section, that study revealed over-activation of NMDA receptors that finally led to cell death [11]. The discrepancies between in vitro and in vivo results highlight the fact that the choice of the model, and indirectly, the dose and the time of exposure are of crucial importance in toxicological studies. 

### 3.3. Physiological Significance of Down-Regulation of the NMDA Receptor Protein Complex by AgNPs/Ag^+^

With the exception of being toxic to neurons while overactivated, NMDA receptors are crucial for neuronal survival and plasticity, particularly during development. Synaptic potentiation (LTP) and synaptic depression (LTD) are two phenomena of NMDA receptor activity-dependent long-term changes in synapses which are believed to underlie learning and memory processes. These phenomena are of considerable interest in the context of the results of the current work.

The precise regulation of expression of ionotropic glutamate receptors underlies the process of maturation of the developing brain. Early in development, the GluN2B regulatory subunit dominates in NMDA receptors and is then gradually replaced by the GluN2A isoform [32], whereas GluN1 is an obligatory component of the heteromeric NMDA receptor. Thus, these subunits were investigated as predominantly expressed in immature excitatory synapses. We found that prolonged 3-week-exposure to a low dose of AgNPs/Ag^+^ significantly decreases expression of both subunits. Multiple recent studies indicate the physiological importance of these NMDA receptor constituents and show that GluN1 and GluN2B knockout mice die perinatally [33]. 

Likewise, during the first few postnatal weeks, NMDA receptors are significantly involved in control of AMPA receptors, which are another type of ionotropic glutamate receptor, by regulating their synaptic content via the LTP/LTD processes [34]. As reported, deletion of the GluN1 and GluN2B alleles during the early postnatal stage disrupts neuronal circuits by dysregulation of synaptic AMPA receptor expression and potentiation of AMPA receptor currents [17]. 

NMDA receptors conduct currents when their glutamate agonist is bound. Simultaneously occurring depolarization and ligand binding trigger maximal calcium influx through the receptor’s channel which further activates LTP/LTD-related molecular signaling cascades [35]. Therefore, when the binding capacity is diminished, disturbances in this process are expected, particularly if expression of some of the PSD-related proteins involved in LTP/LTD signaling pathways decrease at the same time. 

However, a large body of evidence indicates that induction of LTP/LTD is an extremely complex process involving other glutamate receptors, including AMPA receptors (for a review see: [35]). Our results revealed decreased binding of radioactive glutamate in membrane fractions isolated from brains of both AgNP- and Ag^+^-exposed rats that could theoretically also reflect a lower number of binding sites of AMPA receptors. Since there are several different types of glutamate receptors, glutamate may label other receptor populations in addition to NMDA receptors. Therefore, we additionally assessed the binding of MK-801 that is a selective irreversible antagonist of NMDA receptor. The binding of MK-801 was observed to decrease to the similar extent as the binding of radioligand glutamate, suggesting that there is a lower density of NMDA receptors. 

The diversity of signaling proteins implicated in LTP signifies multiple intracellular cascades capable of inducing LTP [34]. A thorough study on related LTP/LTD phenomena was not a goal of the current work. Therefore, we did not examine specifically related molecular pathways. However, stimulation of NMDA receptors results in activation of several downstream pathways, among which GluN2B-PSD-95-nNOS-NO-cGMP is a signaling cascade involved in the LTP formation. NO, acting through its second messenger cGMP, has long been implicated in the generation of LTP and other types of synaptic plasticity, coupling NMDA receptors and the neuronal isoform of NO synthase (nNOS) [36]. Moreover, SynGAP has been also shown to be involved in synaptic plasticity [37].

Alterations in LTP/LTD formation have been shown to be implicated in various brain diseases such as Alzheimer’s disease (AD), depression, and anxiety disorders [38]. For example, in AD, toxic soluble Aβ oligomers decrease the expression of synaptic NMDA receptors thereby contributing to impairment of LTP [39]. It is possible that similar mechanisms take place during exposure to AgNPs/Ag^+^. AgNP-induced decreased expression of the main components of the NMDA receptor complex may lead to weakening of LTP-related synaptic plasticity during development. It is fortunate that the early changes observed in excitatory synapses are not long-lasting changes. At two months post-exposure (PND 90), essentially all of the examined parameters return to control levels, except for expression of PSD-95 which remains at a lower level. 

Thus, the delayed response to the presence of silver is not related to the downregulation of the examined genes and inhibition of the signaling pathway, the activity of which is likely normalized as indicated by the normal level of cGMP. The observed decrease in the level of PSD-95 protein may be a consequence of direct silver–protein interactions, particularly with the -SH groups. This may change the spatial structure of protein and thereby the response to the antibody used in W-B. 

In considering the similarities and differences in the mechanisms of particulate vs. ionic silver-evoked toxicity, we compared the effects of both silver forms. We found that they are both absorbed in the gastrointestinal tract to a similar extent and deposited in brain tissue over time, although the concentration of silver is significantly higher in brain tissue of Ag citrate-exposed rats relative to AgNP-exposed animals. However, expression of proteins related to the NMDA receptor complex is similar in both groups. 

In conclusion, the results of this work provide a basis for characterization of the developmental impact of AgNPs/Ag^+^ on the glutamatergic NMDA receptors. Our findings indicate that NMDA glutamate receptor is a target of both forms of silver. AgNP/Ag^+^-induced neurotoxicity in immature rat brain is associated with down-regulation and a decrease in density of NMDA receptors. Expression profiles of abundant constituents of the NMDA receptor complex together with ultrastructural characteristics and receptor binding assay suggest that AgNPs/Ag^+^ may induce disintegration of the glutamatergic synapses, potentially leading to the observed disturbed molecular pattern of synaptic plasticity. 

The results significantly broaden our knowledge of the influence of AgNPs on developing organisms, pointing out the potentially dangerous consequences of unlimited use of nanoparticulate silver in products developed for infants and children.

## 4. Materials and Methods

### 4.1. Silver Nanoparticles 

AgNPs as a colloidal solution of nanoparticles 10 ± 4 nm in diameter, stabilized in sodium citrate, obtained from Sigma-Aldrich (CAS No.730785) were used throughout the study. Sodium citrate prevents agglomeration of nanoparticles, thereby maintaining their dispersed state. Analysis of the degree of dispersion and size distribution of purchased AgNPs was performed as described previously [40].

Silver citrate (Ag citrate) was used as positive control for estimating the results of the ionic form of silver (Ag^+^). 

### 4.2. Animals and Experimental Design

Experimental procedures using animals were carried out in strict accordance with the EU Directive for the Care and Use of Laboratory Animals (Directive 2010/63/EU) and in compliance with the ARRIVE guidelines. All procedures were approved by the Local Experimental Animal Care and Use Committee in Warsaw (488/2017). 

The study was conducted on two-week-old Wistar rat pups of both sexes purchased from the Animal House of the Mossakowski Medical Research Institute, Polish Academy of Sciences (Warsaw, Poland). Pups at postnatal day 14 (PND 14) were randomly allocated into three groups (*n* = 38 pups per group): i) an experimental group treated with AgNPs, ii) a positive control group treated with Ag citrate (as a source of Ag ions), and iii) a negative control group treated with saline. Appropriate solutions were administered once daily by oral gavage at a dose of 0.2 mg/kg body weight (b.w.)/day for 21 consecutive days. All analyses were performed early (at postnatal day 35; PND 35) and late (PND 90) after exposure in order to assess early and late effects of exposure, respectively. 

Animals were sacrificed by decapitation, forebrains were quickly removed and frozen at −80 °C for further assays. For microscopic analyses, animals were anesthetized and perfused (details described below). The number of animals taken for each analysis is indicated in the respective figure legends.

Blood and brain samples were collected at 35 PND and 90 PND and sent for measurement of silver concentration to the certified Laboratory (“ZdroChem” Sp. Z o.o., Biological and Chemical Research Centre, University of Warsaw, Warsaw, Poland). The analysis of silver concentrations was performed using inductively coupled plasma mass spectrometry (ICP-MS; Elan 6100 DRC Sciex Perkin Elmer, Markham, Ontario, Canada).

### 4.3. Ultrastructural Analysis of Synapses in Brain Samples

After deep anesthesia with Nembutal (80 mg/kg b.w.), the animals were perfused initially with 0.9% NaCl in 0.01 M sodium-potassium phosphate buffer pH 7.4 and subsequently with 2% paraformaldehyde and 2.5% glutaraldehyde in 0.1 M cacodylate buffer, pH 7.4. Brain samples of all rat groups were collected, fixed in the above ice-cold fixative solution and post-fixed in 1% OsO4 solution. The material was then subjected to the routine method of staining for transmission electron microscopy (TEM) analysis. The material was dehydrated in the ethanol gradient and embedded in epoxy resin (Epon 812). Furthermore, ultra-thin sections (60 nm) were stained with 9% uranyl acetate and lead nitrate and examined by TEM (JEM-1200EX, Jeol, Japan). 

### 4.4. Immunohistochemical Procedure and Microscopic Analysis 

After anesthesia with a lethal dose of Vetbutal, rats were perfused through the heart with phosphate-buffered saline (PBS) and 4% paraformaldehyde in PBS. Brains were isolated, cryoprotected in increasing concentrations of sucrose solutions (10%—overnight, 20%—2 days, 30%—5 days), frozen and cut into 20-μm-thick sections. Sections were stained with primary anti-NMDA receptor 1 antibody (1:50; Cell Signaling; Cat. No. 5704) or anti-NMDA receptor 2B antibody (1:50; Proteintech; Cat. No. 21920-1-AP) and co-stained with mouse primary anti-MAP2 (1:750; Sigma Aldrich; Cat. No. M4403). Subsequently, the secondary goat anti-mouse antibodies conjugated with Alexa Fluor 488 (1:500; Invitrogen; Cat. No. A 21121) and the secondary goat anti-rabbit antibodies conjugated with Alexa Fluor 546 (1:500; Molecular Probes Invitrogen; Cat. No. A11010) were added and the slides were exposed for 60 minutes in the dark. Cell nuclei were stained with Hoechst 33258 (1 μg/mL; Sigma-Aldrich; Cat. No. B2261). Control of immunostaining specificity was established by the omission of the primary antibodies in the incubation mixture. The slides were examined using the LSM 780/ELYRA PS.1 super-resolution confocal system (Carl Zeiss, Jena, Germany). Mean fluorescence intensity on micrographs was measured using ZEN 2.6 software (Carl Zeiss, Jena Germany). 

Microscopic analysis was performed in Laboratory of Advanced Microscopy Techniques, Mossakowski Medical Research Institute, Polish Academy of Sciences, Warsaw, Poland.

### 4.5. Western Blot Analysis

The brain tissues (forebrains) were homogenized in 50 mM phosphate buffer pH 7.4, containing 10 mM EGTA, 10 mM EDTA, 0.1 mM PMSF and 100 mM NaCl in the presence of a protease inhibitor cocktail (1 µg/mL leupeptin, 0.1 µg/mL pepstatin and 1 µg/mL aprotinin). Equal protein samples were mixed with loading buffer and subjected to 10% SDS-polyacrylamide gel electrophoresis, and then transferred to nitrocellulose membrane. The membrane-bound proteins were then immunostained with primary antibodies for anti-NMDA receptor 1 (1:500; Sigma Aldrich, Poznań, Poland; Cat. No. SAB4501302), anti-NMDA receptor 2B (1:500; Proteintech; Cat. No. 21920-1-AP), anti-nNOS (1:500; Sigma Aldrich; Cat. No. SAB4502010), anti-SynGAP (1:1000; Abcam; Cat. No. ab3344), anti-PSD95 (1:2000; Invitrogen; Cat. No. 6G6-1C9) and anti-actin (0.5 µg/mL; Abcam; Cat. No. ab 3280). Next, a secondary antibody conjugated with HRP was applied (1:5000; Sigma-Aldrich; Cat. No. A-9169). The tagged proteins were detected using the chemiluminescence ECL kit. The films were scanned using ImageScanner III (GE Healthcare, LabScan 6.0 Freiburg, Germany) and quantified using the Image Quant TL v2005 program.

### 4.6. Analysis of Gene Expression by qPCR

After decapitation, the forebrain samples were isolated at PND 35 or PND 90 under sterile conditions, placed in liquid nitrogen and stored at −80^◦^C for further analysis. 

Total RNA was extracted from the brain samples using TRI Reagent (Sigma-Aldrich, St. Louis, MO, USA) according to the method of Chomczyński and Sacchi [41]. The purified RNA was quantified using a DS-11Fx nano-spectrophotometer/fluorometer (De Novix, Wilmington, DC, USA). 

cDNA was synthesized from 2 µg of total RNA by a reverse transcription kit (Life Technologies, Forest City, CA, USA). The RT-PCR conditions included reverse transcription 42 °C for 45 min followed by denaturation at 94 °C for 30 s. Quantitative real time PCR analysis was performed using TaqMan assays. The primers specific for rat were as follows: for PSD 95 assay ID: Rn00571479_m1, for Syngap assay ID: Rn00710435_m1, for nNOS assay ID: Rn00583793_m1, for NMDA receptor 1 assay ID: Rn 01436034_m1, and for NMDA receptor 2B assay ID: Rn 00680474_m1 (Life Technologies, Forest City, CA, USA). Analysis was conducted on a Roche LightCycler® 96 system, using 5 μL of RT product, TaqMan PCR Master Mix, primers and TaqMan probe in a total volume of 20 μL under the following conditions: initial denaturation at 95 °C for 10 min, 50 cycles of 95 °C for 15 s and 60 °C for 1 min. Each sample was analyzed in triplicate. The relative levels of mRNA expression of examined genes were normalized to actin (Actb) as an internal control and calculated using the _ΔΔ_Ct method.

### 4.7. Preparation of Membrane Fractions and Receptor Binding Assay 

A crude cortical membrane fraction was isolated from the forebrains of the control and silver-treated rats according to the method of Reynolds [42,43]. The experimental procedure was performed as described previously in detail [44] with minor modifications. The brain tissue was homogenized in buffer containing 0.32 M sucrose, 20 mM HEPES and 1 mM EDTA (pH 7.4) using a Polytron mechanical homogenizer. The membrane suspension was then centrifuged at 40,000× *g* for 10 min in 4 °C using a Beckman Coulter Optima XNP-100 ultracentrifuge (Beckman Coulter Inc., Brea, CA, USA). The supernatant was decanted, and the pellet was resuspended in 15 mL of an isolation buffer and homogenized again. The homogenization and centrifugation procedures were repeated three times and the samples were stored at −80 °C for two weeks. 

Prior to each binding experiment, the frozen pellets were thawed and washed twice in 20 mM HEPES buffer (pH 7.4) with 1 mM EDTA, and twice in HEPES buffer without EDTA in order to remove endogenous amino acids during ultracentrifugation (40,000× *g* for 10 min). The assay tubes contained the sample of cell membranes (200 µg of protein per tube) and the radioligands 4 nM [^3^H] glutamate (as endogenous agonist of glutamate receptors) or 4 nM [^3^H] MK-801 (as the specific irreversible antagonist of NMDA receptors) in order to determine total binding sites (TBS) of glutamate receptors and NMDA receptors, respectively. Non-specific binding (NSB) was determined in the presence of 10 µM unlabeled glutamate or MK-801. The specific binding (SB) of [^3^H] glutamate or [^3^H] MK801 was calculated as the difference between total and non-specific binding (SB = TBS – NSB) for each radioligand. The samples were incubated at room temperature for 30 min on a shaker and mixed at a frequency of 240 x/min. The incubation was terminated by rapid filtration on Whatman GF/B filters (Whatman International Ltd., Maidstone, England) using a Brandel MPR-24 Cell Harvester (Labequip, Markham, ON, Canada). Radioactivity was measured by liquid scintillation spectrometry using a Wallac 1409 Counter. The assays were performed in triplicate for individual rats. 

### 4.8. cGMP Assay

To measure cGMP levels, the frozen forebrains were homogenized on ice in cold 5% trichloroacetic acid (TCA). Homogenates were centrifuged at 1500× *g* for 10 min at 4 °C and the supernatants were collected. The TCA was extracted from the sample using water-saturated ether. The level of cGMP was determined using a cyclic GMP Elisa kit (Cayman Chemical, No 581021) according to the manufacturer’s instructions. The plate was read with a wavelength of 410 nm using a microplate reader (FLUOstar Omega, BMG Labtech, Birkerød, Germany). The concentration of cGMP was expressed as pmol per g of wet tissue.

### 4.9. Statistical Analysis

The results are presented as means ± SD from the number of experiments indicated in figure legends. One-way analysis of variance (ANOVA) followed by Tukey’s multiple comparisons test was applied to assess differences between groups with *p* level < 0.05 considered as significant. The data analyses were performed using GraphPad Prism Software, version 6.0 (GraphPad Software, La Jolla, San Diego, CA, USA). 

## Data Availability

The data presented in this study are available on request from the corresponding author.
